# Lyophilized pathogen-inactivated platelet lysate enriched hydrogel for cartilage tissue engineering

**DOI:** 10.3389/fbioe.2026.1986446

**Published:** 2026-10-27

**Authors:** Gabriele Boretti, Hanna Tiainen, Anna Marczak, Helena Hamzehpour, Stina Simonsson, Paolo Gargiulo, Ólafur Eysteinn Sigurjónsson

**Affiliations:** 1 School of Science and Engineering, Reykjavik University, Reykjavik, Iceland; 2 The Blood Bank, Landspitali – The National University Hospital of Iceland, Reykjavik, Iceland; 3 Department of Prosthetic Dentistry and Oral Function, Institute of Clinical Dentistry, University of Oslo, Oslo, Norway; 4 Faculty of Pharmaceutical Sciences, University of Iceland, Reykjavik, Iceland; 5 Department of Clinical Chemistry and Transfusion Medicine, Institute of Biomedicine, University of Gothenburg, Gothenburg, Sweden

**Keywords:** cartilage, hydrogel, platelet lysate, regenerative medicine, tissue engineering

## Abstract

Platelet-derived products are increasingly investigated for cartilage regeneration because of their high content of bioactive factors. While rapid clearance after direct injection has been partly addressed by hydrogel encapsulation, many platelet-enriched hydrogels still rely on variably processed platelet derivatives, leaving challenges in standardization and storage stability. Here, we developed a gelatin-based hydrogel enriched with lyophilized, pathogen-inactivated human platelet lysate (L-PIPL) as an off-the-shelf platelet-derived reservoir for localized delivery of chondrogenic cues. L-PIPL was produced from expired pathogen-inactivated platelet units by freeze–thaw lysis and lyophilization, then incorporated into a gelatin-based hydrogel at 5, 10, and 15% (w/v). Composite hydrogels were characterized by scanning electron microscopy (SEM), attenuated total reflectance Fourier-transform infrared spectroscopy (ATR-FTIR), rheology, transforming growth factor beta 1 (TGF- β 1) release, and soluble protein analysis. Cytocompatibility and early chondrogenic response were assessed in extruded, crosslinked cell-laden constructs using C20A4 chondrocytes and adipose-derived mesenchymal stromal cells (AD-MSCs). L-PIPL preserved the core hydrogel chemistry while modulating microarchitecture and viscoelastic properties. Moderate enrichment, particularly 5%–10% (w/v), maintained gel-like behavior and cytocompatibility, whereas 15% L-PIPL reduced network stability and cell viability. Release of bioactive factors was dose-dependent, with an initial burst followed by slower release over time. In AD-MSC-laden constructs, L-PIPL increased metabolic activity, with the 10% formulation showing the highest activity, and the 5% formulation showing the most favorable early chondrogenic commitment. L-PIPL-enriched hydrogels provide a standardized, cytocompatible, and bioactive matrix for localized delivery of platelet-derived factors. Overall, 5% L-PIPL emerged as the most promising formulation for further development in cartilage tissue engineering.

## Graphical Abstract

**Figure:**
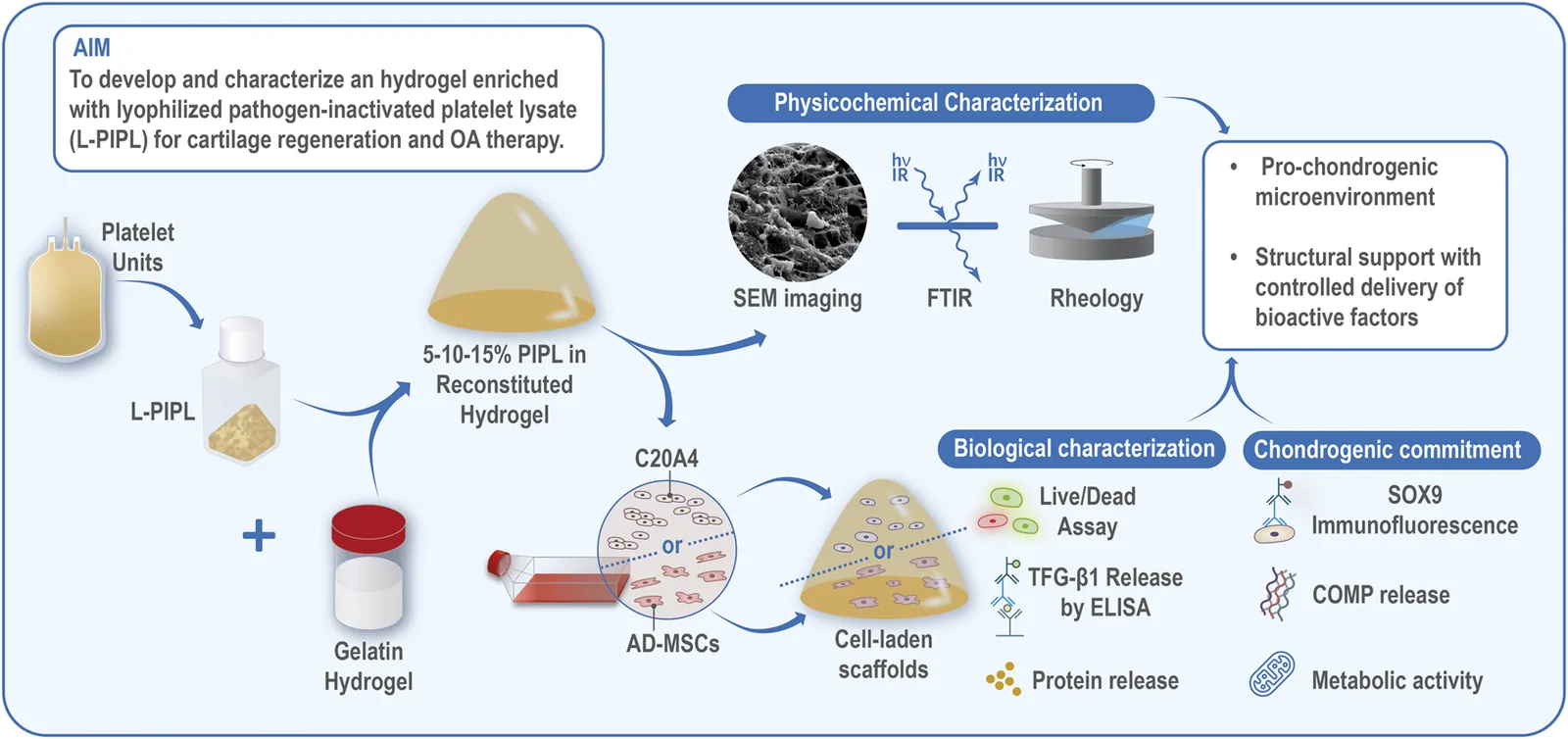
A gelatin-based hydrogel enriched with lyophilized, pathogen-inactivated platelet lysate (L-PIPL) serves as an injectable/printable bioactive scaffold that combines mechanical support with controlled cytokine release for cartilage repair. Cell-laden constructs were evaluated biologically, while acellular scaffolds underwent physicochemical characterization.

## 1 Introduction

Articular cartilage is a specialized connective tissue essential for joint integrity and movement, characterized by a highly organized extracellular matrix (ECM) with limited intrinsic repair capacity Buckwalter and Mankin (1997); Sophia Fox et al. (2009). Degeneration of cartilage is one of the main drivers of osteoarthritis (OA), a disease with a lifetime risk approaching 45% and a growing global prevalence Losina et al. (2013); Neogi (2013); Scheuing et al. (2023). Current clinical strategies for osteoarthritis primarily provide symptomatic relief through pharmacological or physical interventions. However, they do not address the degeneration of articular cartilage. In advanced stages, when joint damage becomes extensive and function is severely impaired, surgical options such as joint replacement are often required. Although these approaches can palliate pain and transiently improve function, they do not restore the structure of the native cartilage or halt disease progression Huey et al. (2012); Armiento et al. (2019). Consequently, there remains an urgent need for regenerative strategies that directly target the underlying loss of healthy cartilage tissue, aiming not only to alleviate pain but also to restore joint structure and prevent the need for surgical intervention.

Regenerative medicine has emerged as a promising field for addressing this unmet clinical need. Cell-based or cell-instructive cartilage tissue engineering strategies have become a major focus for the treatment of focal chondral defects and early degenerative changes Skoracka et al. (2024); Migliorini et al. (2025); however, intra-articular stem cell injections alone do not achieve complete joint repair, highlighting the need for approaches that combine biological and structural support Lin et al. (2025); Stampoultzis et al. (2021). To address these challenges, manufacturing approaches such as three-dimensional (3D) bioprinting have attracted interest in cartilage tissue engineering. This technology enables the precise spatial arrangement of biomaterials, cells, and bioactive factors, facilitating the fabrication of complex, tissue-mimicking constructs. Biomaterials, particularly hydrogels, are central to this approach due to their high water content, biocompatibility, and tunable mechanical properties, which can mimic the native ECM and support chondrogenic differentiation Tong et al. (2022); Yang et al. (2022); Nguyen et al. (2017). Among available hydrogel classes, those that resemble the healthy cartilage ECM, such as gelatin-based systems, alginate, and hyaluronic acid, are most commonly used Boretti et al. (2025). Beyond their use as printed scaffolds, hydrogels can be injected *in situ* to provide mechanical support and serve as depots for the controlled release of pro-regenerative molecules, complementing or replacing direct injections Liu et al. (2017); Zhu et al. (2022); Chen et al. (2025).

Platelet-derived products, including platelet-rich plasma (PRP) and platelet lysate (PL), are attractive bioactive additives for cartilage repair Sánchez et al. (2022); Liau et al. (2021); Zhang J.-Y. et al. (2024); Valtetsiotis et al. (2024). They contain high concentrations of growth factors such as platelet-derived growth factor (PDGF), transforming growth factor-beta (TGF- β ), and epidermal growth factor (EGF), which regulate cell proliferation, matrix synthesis, immunomodulation, and tissue remodeling. Although both are platelet-derived products, PRP and PL represent distinct biological preparations: PRP contains concentrated intact platelets, whereas PL is generated by disrupting platelets to release their bioactive contents, followed by removal of platelet debris. Nevertheless, direct intra-articular delivery of PL is constrained by rapid clearance and uncontrolled release kinetics Wu et al. (2022); Gupta and Maffulli (2024). Encapsulating platelet derivatives within hydrogels is therefore an appealing strategy to achieve localized, sustained release of regenerative cues Zhang P. et al. (2024); Russo et al. (2016); Jooybar et al. (2019); Cao et al. (2025); Tang et al. (2021). As a human, xeno-free product, platelet lysate also mitigates immunogenic and regulatory concerns relative to animal-derived supplements Burnouf et al. (2023); Jonsdottir-Buch et al. (2013).

A major barrier to clinical translation, however, is the lack of standardized methods for PL production, quality control, and storage. Addressing these issues could improve batch-to-batch consistency, safety, and logistical feasibility for routine clinical use. Recent studies have focused on this topic, outlining prevalent production methods and storage conditions De Korte et al. (2024). Compared with fresh PL or PRP, lyophilized, pathogen-inactivated platelet lysate (L-PIPL) couples validated pathogen reduction with dry-state stability, supporting long-term storage, room-temperature transport, and simple point-of-care reconstitution Wendland et al. (2025). These features make L-PIPL particularly suitable for integration into gelatin-based hydrogels, enabling standardized dosing and on-demand preparation in translational settings.

In this study, we present the development and characterization of a cartilage-oriented hydrogel enriched with lyophilized, pathogen-inactivated platelet lysate (L-PIPL). The L-PIPL used here was produced from pooled, pathogen-inactivated platelet concentrates through a standardized Blood Bank workflow and subsequently lyophilized before incorporation into the hydrogel. The primary application investigated was a bioactive cell-laden matrix combining structural support with platelet-derived biochemical cues. Specifically, we (i) formulate and characterize an extrudable L-PIPL hydrogel that provides both structural support and sustained release of platelet-derived factors, and (ii) assess its cytocompatibility and early chondrogenic potential *in vitro*. We hypothesize that L-PIPL enrichment will convert a gelatin-based hydrogel into a tunable bioactive matrix capable of delivering platelet-derived factors in a dose-dependent manner, while maintaining structural and rheological properties compatible with cell encapsulation and supporting early chondrogenic responses.

## 2 Materials and methods

To investigate the effects of enriching a gelatin-based bioink with lyophilized, pathogen-inactivated platelet lysate (L-PIPL), we used the commercially available GelXA Cartilage bioink (CELLINK, Gothenburg, Sweden). This bioink is composed of gelatin methacryloyl (GelMA), sodium alginate, xanthan gum, hyaluronic acid methacrylate (HAMA), and laminin-521, a mix of biomaterials made to mimic the cartilage extracellular matrix (ECM). Hyaluronan and laminin are included to enhance cytocompatibility and support chondrogenic differentiation, while the methacrylated components enable photo-crosslinking initiated by lithium phenyl-2,4,6-trimethylbenzoylphosphinate (LAP), while alginate permits ionic crosslinking with calcium chloride (CaCl_2_). The nominal degrees of methacrylation for GelMA (45%–55%) and HAMA (15%–25%) are provided by the manufacturer.

### 2.1 Lyophilized platelet lysate production

Lyophilized platelet lysate was produced from expired pathogen-inactivated platelet concentrates (PCs) obtained from the Icelandic Blood Bank. Each platelet concentrate was prepared from buffy coats obtained from eight ABO-matched donors and suspended in SSP+ platelet additive solution (Macopharma, Mouvaux, France). PCs manufactured using the same Icelandic Blood Bank workflow have a reported mean platelet concentration of approximately $1.24\times 10^{6}$ platelets/ μ L Delabie et al. (2025). PCs were pathogen-inactivated using the INTERCEPT system (Cerus Corporation, Concord, CA, USA) and stored under continuous agitation at 22 °C for up to 7 days. For this study, PCs bags of approximately 200 mL were frozen at −80 °C on day 8 after collection and stored until a total of 12 units had been accumulated. The use of these materials for research was approved by the National Bioethics Committee (number: VSN19-189). No identifiable donor information was used.

Pathogen-inactivated platelet lysate (PIPL) was generated by subjecting the frozen platelet bags to three freeze–thaw cycles, each consisting of thawing in a 37 °C water bath followed by immediate refreezing at −80 °C (Figure 1a). After the third thawing step, the lysates were pooled irrespective of ABO group into 600 mL bags, increasing the number of donors represented in the final preparation and reducing the influence of individual donor variability. The pooled lysates were centrifuged at 4,975 g for 10 min to remove platelet debris and aggregates. The clarified supernatant was recovered using a plasma extractor, frozen, and subsequently pooled into 1.5 L bags that were stored at −80 °C. All processing steps up to this stage were performed in a closed bag system to preserve product sterility. The pooled PIPL was then processed under laminar flow by filtration through a 0.45 μ m Stericup filter (MilliporeSigma, Burlington, MA, USA), aliquoted, and stored at −80 °C until lyophilization.

**FIGURE 1:**
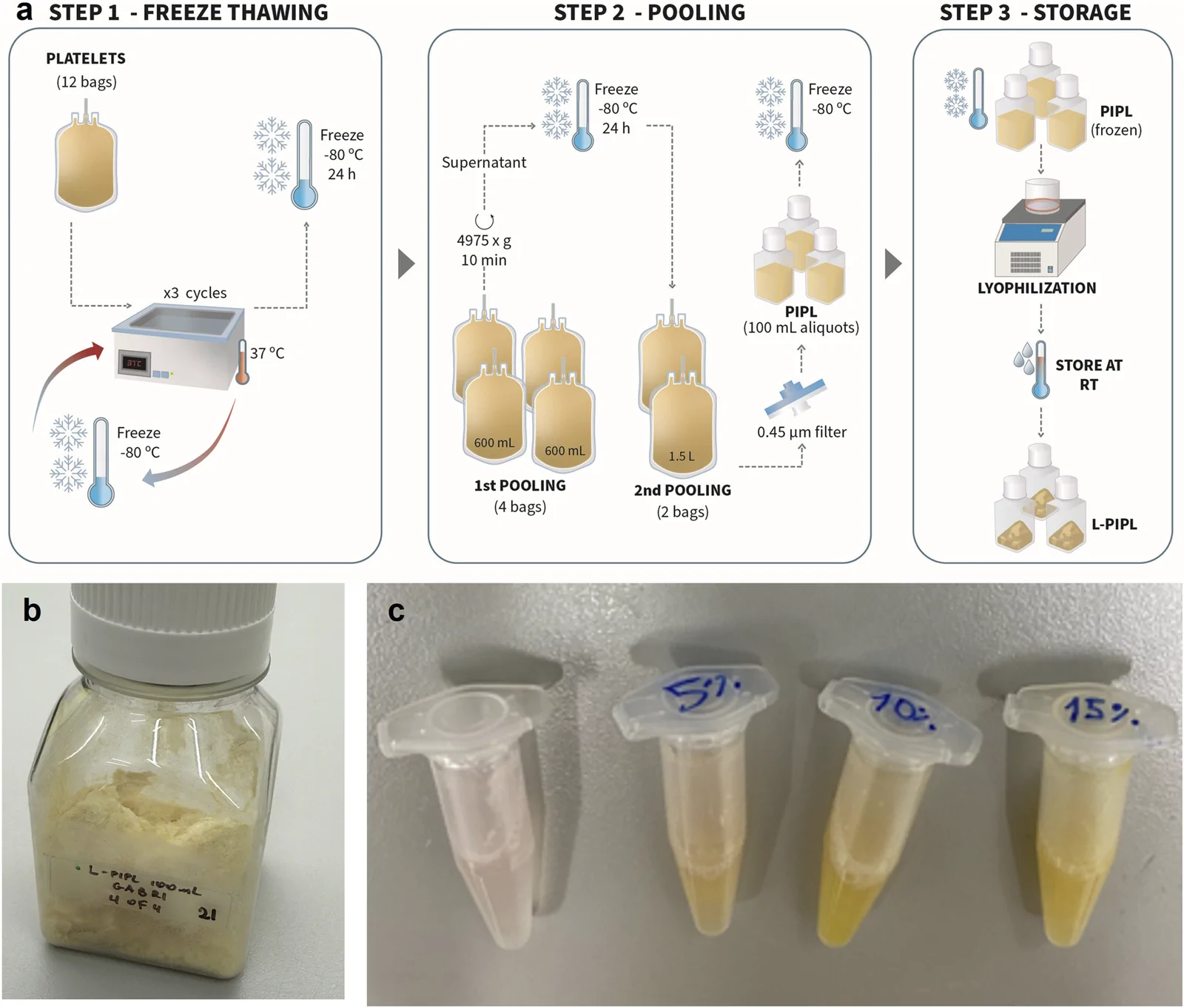
Production of L-PIPL and L-PIPL-enriched hydrogels. **(a)** Schematic representation of the production process of L-PIPL. Expired, pathogen-inactivated platelet concentrates went through freeze-thawing cycles, pooling, filtration, aliquoting, and subsequent freeze-drying. **(b)** Lyophilized, pathogen-inactivated platelet lysate (L-PIPL) powder prior to reconstitution (representative batch). **(c)** Representative Eppendorf tubes of prepared cell-embedded hydrogels with different L-PIPL concentrations. From left to right: control, 5%, 10%, and 15% (w/v) L-PIPL-enriched hydrogel.

Lyophilization of frozen aliquots was performed using a ScanVac CoolSafe freeze-dryer (LaboGene A/S, Allerød, Denmark) for approximately 3 days under a primary drying pressure of 0.04 mbar, with an ice-condenser temperature of −88 ^°^C. The resulting lyophilized product, referred to as L-PIPL, was stored protected from light and moisture at room temperature for up to approximately 2 months before use, consistent with previous reports Chen et al. (2018); Anitua et al. (2021).

### 2.2 Hydrogel preparation and sample fabrication

L-PIPL-enriched hydrogels were prepared at 5%, 10%, and 15% weight/volume (w/v). GelXA bioink was heated to 37 °C and mixed. For each formulation, 0.05, 0.10, or 0.15 g of L-PIPL powder (Figure 1b) was added to 0.95, 0.90, or 0.85 mL of GelXA Cartilage, respectively, under sterile conditions. Blends were gently mixed until homogeneous, resulting in ready-to-use viscous hydrogels shown in Figure 1c. For biological and structural experiments, dome-shaped constructs were formed by cooling the hydrogel to 25 °C and extruding through a 21G blunt syringe needle onto sterile Petri dishes. For confocal imaging, disc-shaped constructs were prepared by extruding 50 μ L of cell-laden hydrogel between two hydrophobic glass slides separated by a 1 mm spacer. The samples were double-crosslinked using 30 s exposure to 405 nm blue light followed by 15 min in a 50 mM CaCl_2_ solution, as previously described Boretti et al. (2024).

### 2.3 Cell cultures

The C20A4 human chondrocyte cell line (Sigma-Aldrich, St. Louis, MO, USA) and human adipose-derived mesenchymal stromal cells (AD-MSCs; StemPro™ Gibco, Thermo Fisher Scientific, Waltham, MA, USA) were used to assess the effect of L-PIPL enrichment. C20A4 cells (passage 5) were expanded in Dulbecco’s modified Eagle medium F-12 (DMEM/F12) supplemented with 10% fetal bovine serum (FBS) and 1% penicillin-streptomycin (P/S). Cells were trypsinized, counted using Trypan Blue, and resuspended at 5 × 10^6^ cells/mL in hydrogel warmed at 37 °C. The cell-laden hydrogel was loaded into a syringe, cooled at 25 °C, and extruded into domes as described. The constructs were incubated in 24-well plates with 2 mL of growth medium, which was replaced every 48 h.

For chondrogenic differentiation, only the hydrogel composites that showed the highest cytocompatibility with C20A4 cells were used. AD-MSCs (passage 6) were expanded in DMEM/F12 medium supplemented with 10% pathogen-inactivated platelet lysate (PIPL) and 1% penicillin-streptomycin (P/S), consistent with previous studies Jonsdottir-Buch et al. (2013), Jonsdottir-Buch et al. (2015). Similarly to C20A4, AD-MSCs were trypsinized, embedded in the hydrogel, loaded into a syringe, and extruded to realize disc-shaped constructs as previously described. Following encapsulation, all AD-MSC constructs were cultured under the same serum-free conditions using StemPro™ Chondrogenesis Differentiation Medium (Gibco, Thermo Fisher Scientific), prepared according to the manufacturer’s instructions. Chondrogenic differentiation was continued for 7 days, with half of the medium replaced every other day.

### 2.4 Physicochemical characterization

#### 2.4.1 SEM imaging

The surface morphologies of the cell-laden hydrogels were explored using scanning electron microscopy (SEM) with a JSM-IT800 microscope (JEOL Ltd., Akishima, Tokyo, Japan). The crosslinked samples were flash frozen in liquid nitrogen to avoid crystal formation and subsequently freeze-dried. To make the biological samples conductive, a layer of gold (12.0 nm thickness) was applied to the surface before investigation. The images were captured with an operating voltage of 20.0 kV and a working distance (WD) ranging from 4.0 to 6.0 mm.

#### 2.4.2 ATR-FTIR analysis

Fourier-transform infrared (FTIR) spectra were collected using a Nicolet iS50 spectrometer (Thermo Fisher Scientific) equipped with an attenuated total reflectance (ATR) module. Uncrosslinked samples were placed directly on the ATR crystal, and spectra were recorded from 4,000 to 400 ${\text{cm}}^{-1}$ . Characteristic bands were evaluated to confirm functional groups and verify the hydrogel composition.

#### 2.4.3 Rheology

Rheology was performed on uncrosslinked samples to mimic pre-deposition flow during extrusion/injection. A Modular Compact Rheometer (MCR; Anton Paar GmbH, Graz, Austria) equipped with planar plates was used for rheological studies with a 1.0 mm gap height. During frequency and amplitude sweeps, a Peltier temperature control hood maintained a constant temperature of 25 °C. An amplitude sweep (frequency 1.0 Hz; strain γ ramped logarithmically from 0.01% to 600%) was first conducted to determine the linear viscoelastic (LVE) region and assess yield/breaking behavior. A frequency sweep (0.1–500 rad/s) was subsequently performed at a strain within the LVE region identified from the amplitude sweep.

### 2.5 Biological characterization

#### 2.5.1 Live/dead viability

C20A4 viability was assessed in cell-laden 3D constructs on days 1 and 5 to evaluate the effects of processing and varying L-PIPL concentrations. Cells were mixed into the hydrogel, followed by extrusion and crosslinking as described above. At each time point, samples were rinsed with phosphate-buffered saline (PBS) and stained for 30 min using the LIVE/DEAD™ Viability/Cytotoxicity Kit (Thermo Fisher Scientific), following manufacturer’s instructions. Confocal images were acquired with OLYMPUS FV1200 (Olympus Corporation, Tokyo, Japan) using a 10 × objective and CellSens software (Olympus). Live/Dead images were processed and quantified using an image-based fluorescence area approach on Fiji (ImageJ), as single-cell counting was limited by cell overlap and spreading within the matrix. For each image, calcein acetoxymethyl ester (calcein AM) and ethidium homodimer-1 (EthD-1) channels were separated, background-subtracted, thresholded using fixed channel-specific thresholds, and converted into binary masks. Calcein-positive area was calculated as the green-positive area normalized to the total image area and used as a measure of live-cell coverage. Mortality rate was calculated as the EthD-1-positive area divided by the sum of calcein-positive and EthD-1-positive areas:

$$Mortality\;rate\;\left(\%\right)=\frac{A_{\text{EthD}-1}}{A_{\text{calcein}}+A_{\text{EthD}-1}}\times 100.$$

At least 5 randomly acquired image fields were quantified per condition and time point.

Furthermore, a Live/Dead assay was performed on AD-MSC-laden hydrogels as a viability quality-control step to confirm stem cell survival following encapsulation and fabrication before subsequent chondrogenic readouts were interpreted. Representative images are reported in Supplementary Figure S1.

#### 2.5.2 TGF- β 1 release

Conditioned medium from C20A4-laden hydrogel cultures was collected after successive intervals of 0.5, 1, 3, 6, 12, 24, and 48 h, corresponding to a final cumulative culture time of 94.5 h, and stored at −80 °C until analysis. Transforming growth factor beta 1 (TGF- β 1) was selected as a representative platelet-derived factor because of its central role in chondrogenic signaling and cartilage matrix formation Patil et al. (2011); Du et al. (2023). Total TGF- β 1 was quantified after activation according to the manufacturer’s protocol using the Human TGF- β 1 DuoSet enzyme-linked immunosorbent assay (ELISA) (R&D Systems, Bio-Techne, Minneapolis, MN, USA). Absorbance at 450 nm with 570 nm background correction was measured on a Multiskan FC plate reader (Thermo Fisher Scientific).

#### 2.5.3 Protein release and metabolic analysis

Conditioned medium was collected every 48 h for the first 6 days from AD-MSC-laden hydrogels cultured in chondrogenic medium. Total protein content was quantified using the Pierce bicinchoninic acid (BCA) Protein Assay Kit (Thermo Fisher Scientific). Absorbance at 562 nm was measured on a Multiskan FC plate reader (Thermo Fisher Scientific). Glucose, lactate, and ionized calcium levels in the medium were evaluated using an ABL800 FLEX gas analyzer (Radiometer Medical ApS, Brønshøj, Denmark).

#### 2.5.4 COMP quantification

Analysis of cartilage oligomeric matrix protein (COMP) release into the medium was performed to evaluate early chondrogenic commitment in a non-destructive way. Medium from AD-MSC-laden hydrogels was collected every 48 h and stored at −80 °C until analysis. Total COMP was quantified according to the manufacturer’s protocol using the Human COMP DuoSet ELISA (R&D Systems, Bio-Techne). Absorbance at 450 nm with 570 nm background correction was measured on a Multiskan FC plate reader (Thermo Fisher Scientific).

#### 2.5.5 Immunofluorescence

AD-MSC-laden hydrogels cultured in chondrogenic medium were collected at days 1, 3, and 7 of chondrogenic differentiation. Briefly, scaffolds were rinsed with PBS, fixed in 10% formalin, and stored at 4 °C until staining. Constructs were then permeabilized with 0.1% Triton X-100 for 40 min and blocked with 10% FBS for 2 h to reduce non-specific binding. Samples were incubated with an Alexa Fluor™ Plus 555-conjugated recombinant mouse monoclonal anti-SOX9 antibody, clone GMPR9 (Invitrogen, Thermo Fisher Scientific), diluted 1:300 in blocking buffer, for 2 h at room temperature on a shaker and then overnight at 4 °C protected from light. Samples were subsequently washed with PBS and incubated with Alexa Fluor™ 647 Phalloidin (Invitrogen, Thermo Fisher Scientific), diluted 1:400 in blocking buffer, for 1 h at room temperature to visualize F-actin. Finally, scaffolds were washed again with PBS and incubated with 4′,6-diamidino-2-phenylindole (DAPI) nuclear counterstain (Invitrogen, Thermo Fisher Scientific; 5 mg/mL stock diluted 1:1000 in PBS) for 15 min at room temperature on a shaker protected from light. Samples were washed with PBS and stored at 4 °C until visualization. Olympus FV1200 confocal microscope was used for acquisition, and images were processed using Fiji (ImageJ).

### 2.6 Statistical analysis

MATLAB R2024b (The MathWorks, Inc., Natick, MA, USA) was used for data processing, plots and statistical analysis. For all quantitative analyses, n=3 independent hydrogel constructs were analyzed per condition. ELISA and BCA measurements were performed in technical triplicates and averaged to obtain one value per construct at each time point. Normal distribution was assessed using the Shapiro–Wilk test. A two-way analysis of variance (ANOVA) was applied to evaluate the effects of *Time* (sampling time points) and *Condition* (L-PIPL concentration), including their interaction. Complete two-way ANOVA results, including F statistics, degrees of freedom, and exact p -values, are reported in Supplementary Table S1.

For graphical annotation of *post hoc* comparisons, pre-planned comparisons were performed independently at each time point. Each L-PIPL-enriched formulation was compared with the corresponding control group at the same time point using Bonferroni-adjusted multiple comparisons. Adjusted p -values below 0.05 were considered statistically significant and are indicated as * p<0.05 , ** p<0.01 , *** p<0.001 , and **** p<0.0001 . Data are reported as mean ± standard deviation (SD).

## 3 Results

The mechanical, chemical and morphological properties of L-PIPL-enriched hydrogels were evaluated to characterize the composite and define an effective loading window for cartilage repair. We first examined the feasibility of hydrogel preparation and extrusion, and then how enrichment affected the hydrogel microstructure, chemistry, and mechanics. Finally, we assessed cytocompatibility, release kinetics of proteins and of a representative chondrogenic cytokine (TGF- β 1), as well as metabolic activity and chondrogenic potential. Together, these data support a moderate L-PIPL loading (5%–10%) as a balanced operating range that boosts bioactivity while preserving structural integrity.

### 3.1 Physicochemical characterization

#### 3.1.1 Morphology and microstructure

Scanning electron microscopy (SEM) was used to evaluate the internal microstructure of the hydrogel samples at increasing L-PIPL concentrations (0%, 5%, 10%, and 15%). As shown in Figure 2, notable differences in porosity and surface architecture were observed depending on the amount of lyophilized platelet lysate incorporated.

**FIGURE 2:**
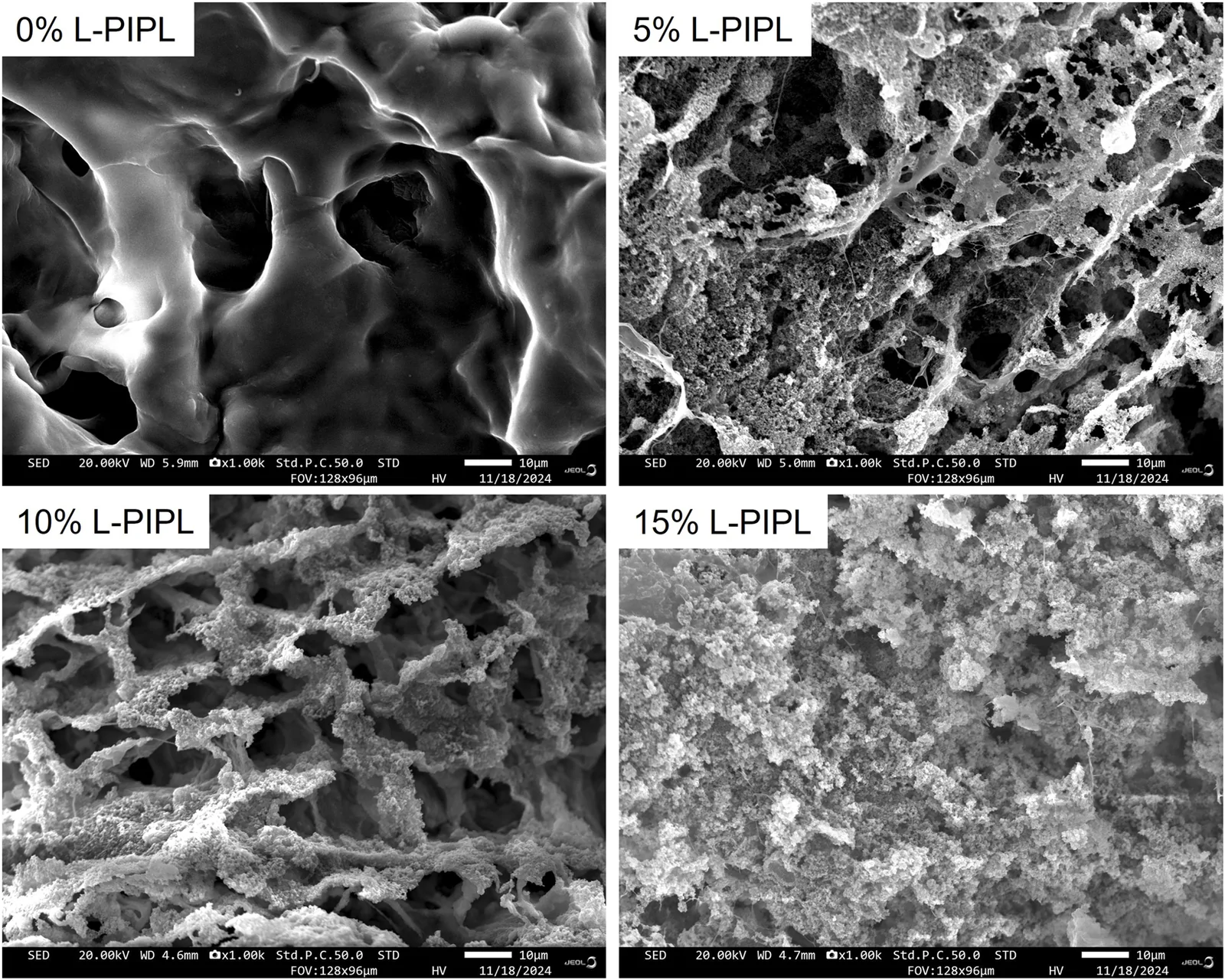
Scanning electron microscopy of crosslinked hydrogels with different L-PIPL concentrations. JEOL JSM-IT800 microscope; 1000 × magnification; scale bar = 10 μm; 20 kV; WD 4–6 mm.

The 0% L-PIPL control exhibited a comparatively smoother matrix with limited pore formation. At 5% L-PIPL, the structure became more heterogeneous, with the emergence of micropores and rougher topography, indicating a possible protein interaction during gelation. At 10% L-PIPL, the hydrogel exhibited a pronounced porous network with interconnected pores and a highly fibrillar morphology, likely favorable for nutrient diffusion and cell infiltration. However, at 15% L-PIPL, the microstructure appeared denser and irregular, with apparent pore collapse or excessive aggregation, potentially caused by saturation of the matrix with particulate content. Overall, SEM analysis demonstrated that moderate enrichment (5%–10% L-PIPL) promoted the formation of an interconnected porous network, while higher concentrations (15%) disrupted the uniformity of the microstructure.

#### 3.1.2 Chemical composition

ATR–FTIR spectra (4,000–400 ${\text{cm}}^{-1}$ ) confirmed that L-PIPL enrichment increased protein-associated signals without altering the base network’s functional groups (Figure 3).

**FIGURE 3:**
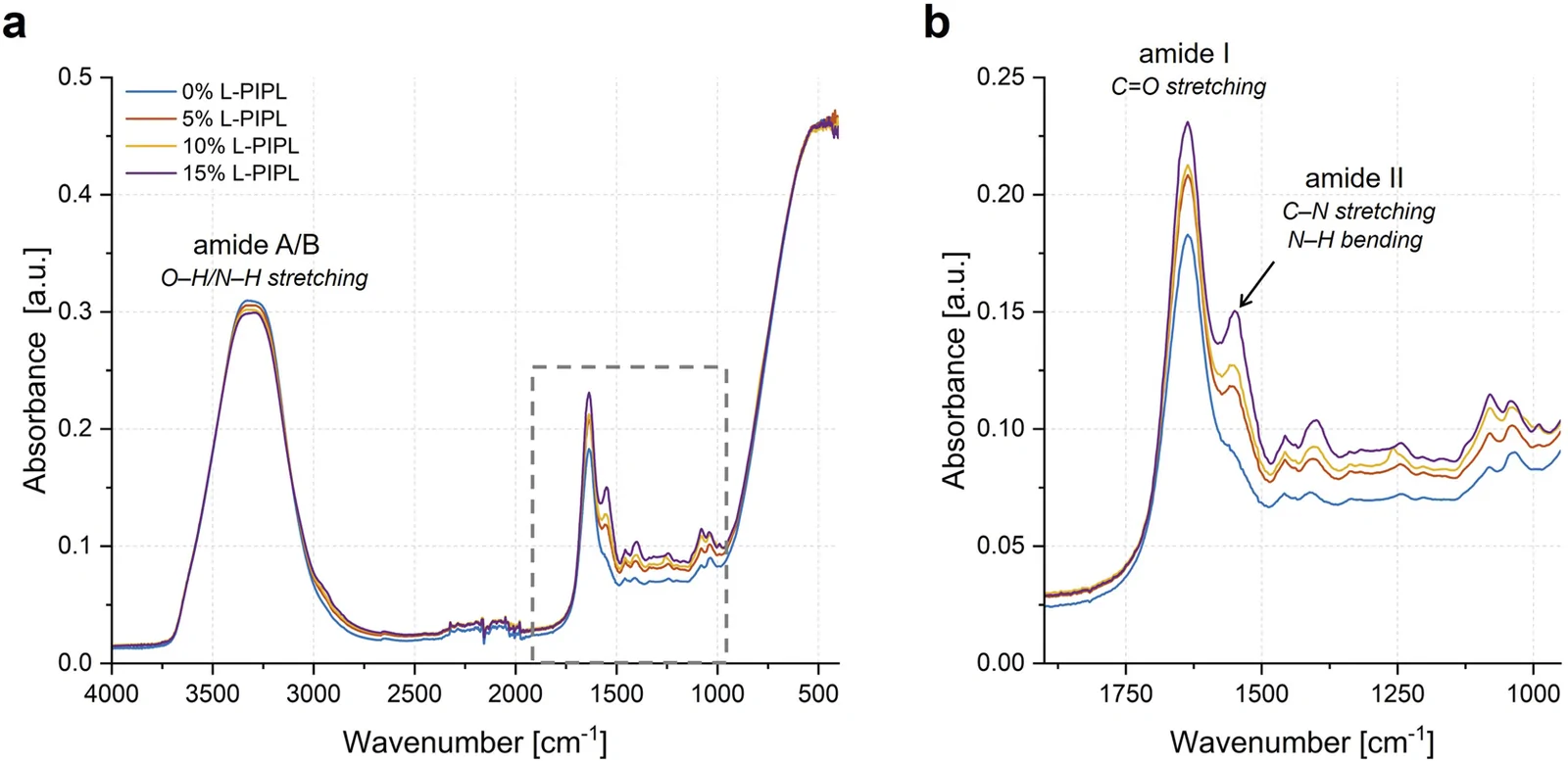
ATR–FTIR spectra of GelXA Cartilage hydrogels with increasing L-PIPL content. **(a)** Full spectra in the 4,000–400 ${\text{cm}}^{-1}$ range. **(b)** Zoom of the fingerprint region highlighting the protein-associated amide I and amide II bands.

All hydrogel formulations, including the control (0% L-PIPL), showed prominent peaks near 3,300 cm^-1^, corresponding to O–H/N–H stretching vibrations. In the fingerprint region, amide I (1650 cm^-1^, C=O stretching) and amide II (1540 cm^-1^, C–N stretching and N–H bending) bands were observed, confirming the presence of proteinaceous components in the GelXA matrix and L-PIPL. The intensity of amide peaks increased with higher L-PIPL concentrations, reflecting the protein-rich nature of the PL. Additional signals were identified in the 1000–1200 cm^-1^ range, corresponding to C–O and C–O–C stretching. No new peaks or shifts in major functional groups were observed with L-PIPL incorporation, indicating that the enrichment process preserved the core chemical structure of the hydrogel network while enhancing its protein content.

#### 3.1.3 Rheology

The viscoelastic properties of L-PIPL-enriched hydrogels were evaluated using amplitude and frequency sweep tests to assess mechanical integrity, strain-dependent network stability, and extrusion-relevant behavior (Figure 4).

**FIGURE 4:**
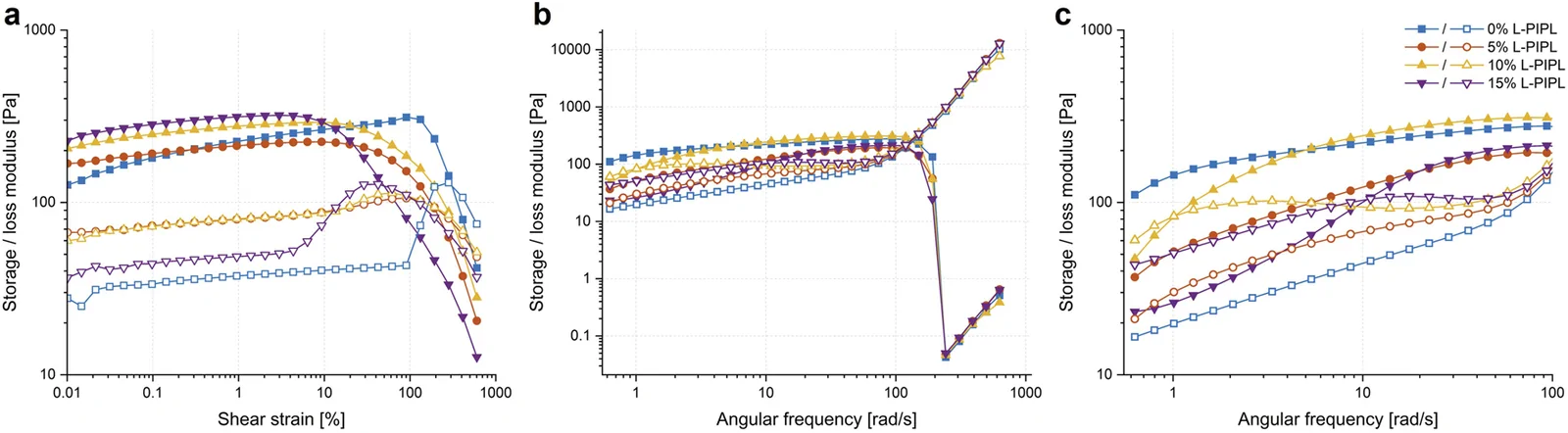
Rheological characterization of uncrosslinked hydrogels (25 °C, 1.0 mm gap). Filled symbols indicate storage modulus $\left(G^{\prime }\right)$ , while open symbols indicate loss modulus $\left(G^{\prime \prime }\right)$ . **(a)** Amplitude sweep at 1.0 Hz as a function of shear strain. **(b)** Frequency sweep over an extended angular-frequency range. **(c)** Magnified frequency sweep in the low-to-intermediate angular-frequency range used to compare the viscoelastic behavior of the hydrogels.

In the amplitude sweep, all formulations displayed gel-like behavior at low strain, with the storage modulus $\left(G^{\prime }\right)$ exceeding the loss modulus $\left(G^{\prime \prime }\right)$ , indicating elastic-dominated responses within the linear viscoelastic region. The incorporation of L-PIPL altered the strain-dependent mechanical response of the hydrogel. At small deformations, the 10% and 15% L-PIPL formulations showed higher $G^{\prime }$ values than the control and 5% samples, suggesting increased initial stiffness upon enrichment. However, increasing L-PIPL content also reduced resistance to large deformation. The 0% hydrogel maintained elastic dominance over a broader strain range, whereas the L-PIPL-enriched hydrogels, particularly the 15% formulation, showed an earlier decrease in $G^{\prime }$ and a more pronounced transition toward network disruption. This behavior indicates that excessive L-PIPL loading increases strain sensitivity and weakens the stability of the uncrosslinked network under high deformation.

Frequency sweep analysis further demonstrated that L-PIPL modulated the elastic–viscous balance in a concentration-dependent manner. In the low-to-intermediate angular-frequency range, the 0% and 5% formulations maintained $G^{\prime }>G^{\prime \prime }$ across most of the tested range, confirming predominantly gel-like behavior. The 10% L-PIPL hydrogel showed a slight viscous contribution at the lowest frequencies, but $G^{\prime }$ rapidly exceeded $G^{\prime \prime }$ as angular frequency increased, and this formulation reached the highest storage modulus at higher frequencies. In contrast, the 15% L-PIPL hydrogel exhibited viscous-dominated behavior at low frequencies, with $G^{\prime \prime }>G^{\prime }$ , followed by a crossover to elastic-dominated behavior at higher frequencies. This suggests that excessive L-PIPL incorporation compromises the slow-deformation stability of the hydrogel network, although elastic behavior is partially recovered under faster oscillatory loading.

Together, these rheological profiles demonstrate that L-PIPL modulates not only the stiffness but also the elastic-viscous balance of the hydrogel in a concentration-dependent manner. While moderate enrichment (5%–10%) enhances mechanical integrity and maintains gel-like behavior, overloading with lysate (15%) results in mechanical brittleness and impairs the rheological performance.

### 3.2 Biological characterization

#### 3.2.1 Live/dead viability

Live/Dead staining was performed on Days 1 and 5 to assess the impact of L-PIPL concentration on C20A4 viability within the hydrogel constructs (Figure 5).

**FIGURE 5:**
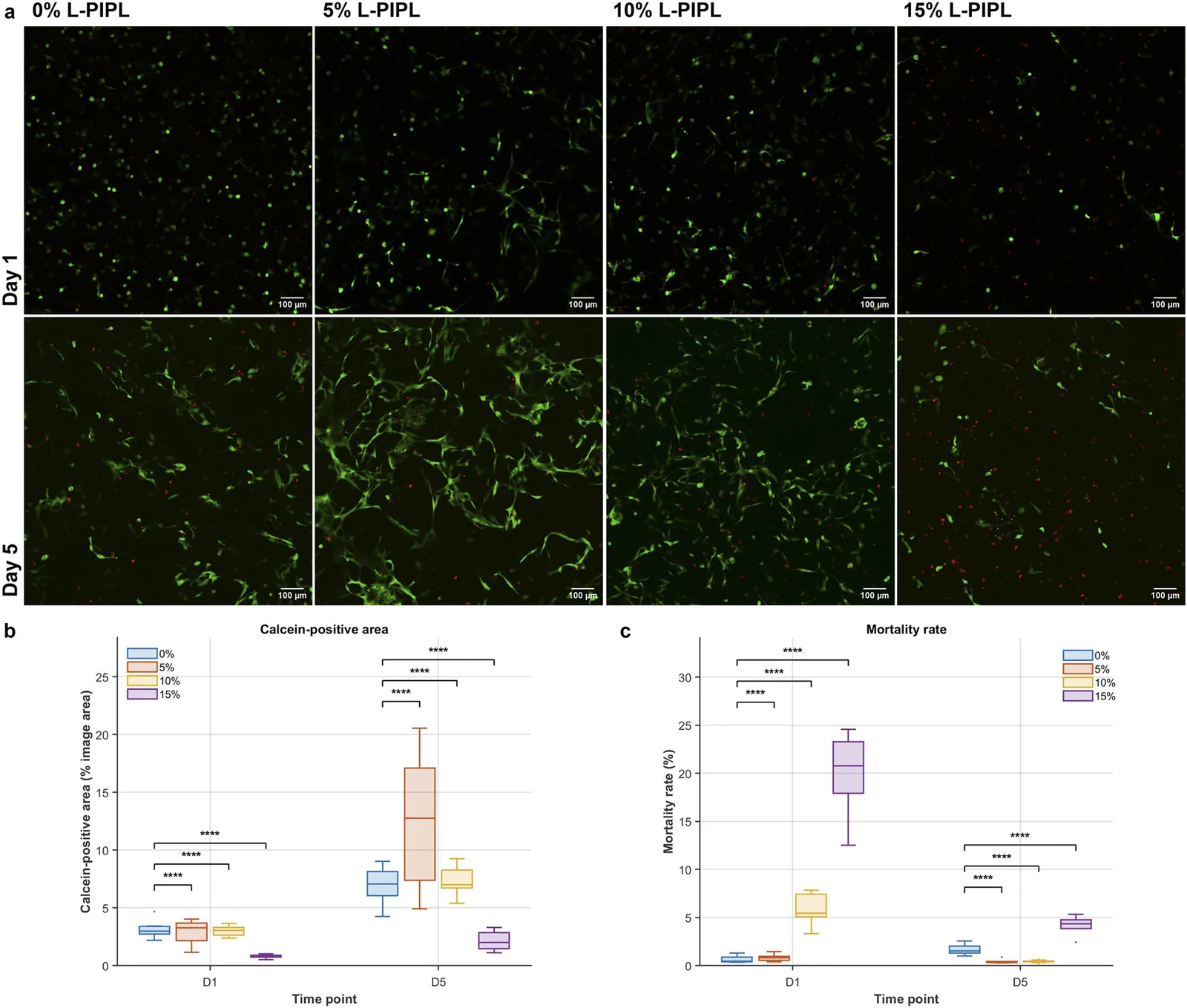
Viability of C20A4 chondrocytes encapsulated in L-PIPL hydrogels at Day 1 and Day 5. **(a)** Representative Live/Dead images showing Calcein AM-positive live cells (green) and EthD-1-positive dead cells (red). Olympus FV1200 microscope; 10 × magnification; scale bar = 100 µm. Quantification of **(b)** calcein-positive area and **(c)** mortality rate. Quantitative data are presented as mean ± SD from at least 5 randomly acquired image fields sampled across three independent hydrogel constructs. Statistical significance was assessed by two-way ANOVA, followed by Bonferroni-adjusted post-hoc comparisons. Asterisks indicate Bonferroni-adjusted comparisons with the 0% L-PIPL control at the corresponding time point: * p<0.05 , ** p<0.01 , *** p<0.001 , **** p<0.0001 .

Greater live-cell coverage was observed in the control and 5% L-PIPL groups, while viability decreased at higher concentrations, particularly in the 15% L-PIPL group. This qualitative trend was supported by image quantification. At Day 1, the calcein-positive area remained low for all groups, with 0%–10% L-PIPL showing comparable values and 15% L-PIPL displaying the lowest signal. Mortality was minimal in the 0% and 5% groups, increased in 10% L-PIPL, and was markedly higher in 15% L-PIPL. By Day 5, the calcein-positive area increased in the 0%–10% groups, reaching the highest values in 5% L-PIPL, while 15% L-PIPL remained significantly lower. Mortality on Day 5 was low in the 5% and 10% L-PIPL hydrogels and remained higher in the 15% L-PIPL group.

On Day 1 embedded cells appeared uniformly distributed with a predominantly rounded morphology. By Day 5 cells exhibited more elongated shapes and denser networks, especially in hydrogels with L-PIPL. However, 15% L-PIPL samples showed reduced viability, with higher red staining and fewer elongated cells.

Overall, these results confirm that the hydrogels are cytocompatible up to 10% L-PIPL enrichment. At 15%, decreased viability may reflect a combination of altered matrix organization and transport within the static 3D culture system together with concentration-dependent biological effects of platelet-derived components.

#### 3.2.2 Growth factor release

TGF- β 1 release into the culture medium was quantified over 4 days to evaluate dose- and time-dependent delivery (Figure 6).

**FIGURE 6:**
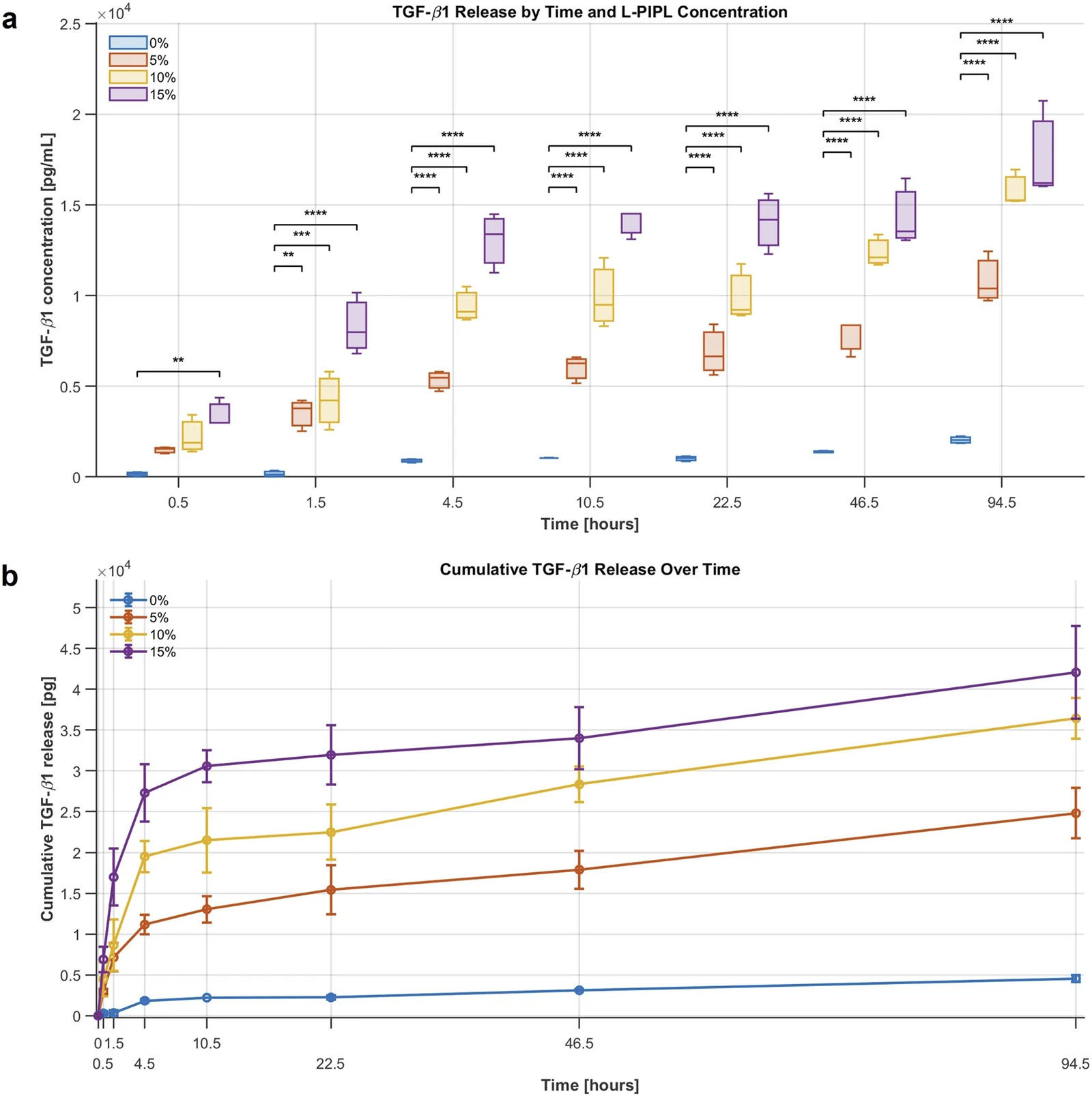
Dose- and time-dependent TGF- β 1 release over 94.5 h ( ≈ 4 days). **(a)** TGF- β 1 concentration in the conditioned medium at each collection time point. **(b)** Cumulative TGF- β 1 release from the cell-laden constructs over time. Quantitative data are presented as mean ± SD ( n=3 independent hydrogel constructs per condition). Statistical significance was assessed by two-way ANOVA followed by Bonferroni-adjusted post-hoc comparisons. Asterisks indicate Bonferroni-adjusted comparisons with the 0% L-PIPL control at the corresponding time point: * p<0.05 , ** p<0.01 , *** p<0.001 , **** p<0.0001 .

While 0% L-PIPL control maintained baseline levels below 200 pg/mL throughout, enriched hydrogels released significantly higher concentrations, with the 15% group reaching around 20,000 pg/mL by the final time point. Cumulative release curves (Figure 6) demonstrated a burst release pattern within the first 4.5 h, especially evident in the 10% and 15% L-PIPL samples. After the initial burst, the release profile plateaued gradually.

These findings confirm that L-PIPL acts as a reservoir of bioactive growth factors and that its incorporation enables a tunable release of TGF- β 1. The dose-dependent profile supports the feasibility of using these hydrogels as delivery vehicles for regenerative signaling in cartilage repair applications.

#### 3.2.3 Metabolic activity, calcium levels and protein release

Metabolic activity and soluble protein content were assessed in conditioned medium collected from AD-MSC-laden hydrogels during the first 6 days of chondrogenic commitment. Glucose levels decreased in a concentration-dependent manner, indicating greater glucose consumption in L-PIPL-enriched hydrogels compared with the control formulation (Figure 7a). Consistently, lactate levels increased with L-PIPL enrichment, with the highest values observed in the 10% L-PIPL group, particularly at days 4 and 6 (Figure 7b).

**FIGURE 7:**
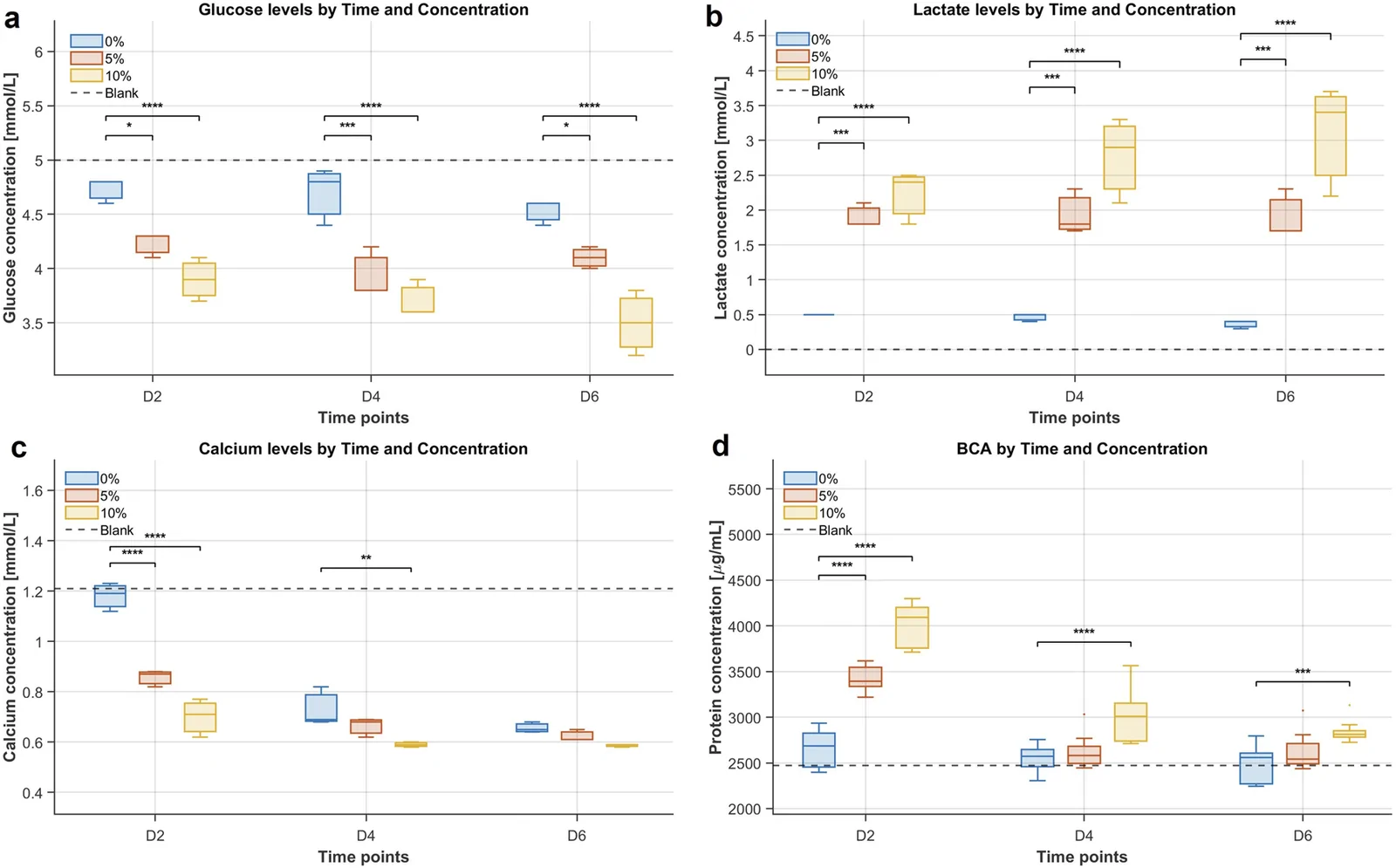
Metabolic activity, ionized calcium levels, and soluble protein content of AD-MSC-laden hydrogels during the first 6 days of chondrogenic commitment. **(a)** Glucose concentration in conditioned medium. **(b)** Lactate concentration in conditioned medium. **(c)** Ionized calcium concentration in conditioned medium. **(d)** Total soluble protein content quantified by BCA assay. The dashed line indicates the blank medium baseline. Quantitative data are presented as mean ± SD ( n=3 independent hydrogel constructs per condition). Statistical significance was assessed by two-way ANOVA followed by Bonferroni-adjusted post-hoc comparisons. Asterisks indicate Bonferroni-adjusted comparisons with the 0% L-PIPL control at the corresponding time point: * p<0.05 , ** p<0.01 , *** p<0.001 , **** p<0.0001 .

Ionized calcium levels in the conditioned medium decreased over time and were lower in L-PIPL-containing groups (Figure 7c). Because the experimental system contains several components capable of influencing calcium availability, such as citrate-containing SSP+ originating from the platelet concentrates and CaCl_2_ used for ionic crosslinking, these measurements were interpreted as reflecting the combined ionic environment of the hydrogel culture system rather than cellular calcium utilization alone.

Total soluble protein content, measured by BCA assay, was highest at day 2 and increased with L-PIPL concentration, with the 10% L-PIPL formulation showing the greatest protein content (Figure 7d). Protein levels decreased at later time points, approaching the blank medium baseline, although L-PIPL-enriched groups generally remained higher than the control. Together, these findings indicate that L-PIPL enrichment increases the availability of proteins and is associated with enhanced metabolic activity of encapsulated AD-MSCs during early chondrogenic culture.

#### 3.2.4 Chondrogenic commitment

Early chondrogenic commitment of AD-MSC-laden hydrogels was evaluated by immunofluorescence staining and COMP quantification in the conditioned medium. At day 1, cells appeared predominantly rounded in all formulations, with limited spreading and relatively low cytoskeletal organization (Figure 8). By day 3, L-PIPL-enriched hydrogels showed increased cell spreading and more evident cytoskeletal organization than the 0% control, particularly in the 5% and 10% formulations. This effect became more pronounced by day 7, where cells in the L-PIPL-enriched hydrogels formed denser and more interconnected cellular networks, while cells in the control hydrogel remained more sparsely distributed and predominantly rounded. SOX9-positive signal was detected at days 3 and 7 and appeared most prominent in the 5% L-PIPL formulation. Separate images for the SOX9 channel are reported in the Supplementary Materials. Consistent with immunofluorescence results, COMP levels in the conditioned medium increased over time and were markedly higher in L-PIPL-enriched hydrogels compared with the control. The 5% L-PIPL formulation showed the highest release of COMP on days 4 and 6, while the 10% formulation also showed higher concentrations of COMP than the control. These findings suggest that L-PIPL enrichment promotes early chondrogenic commitment of encapsulated AD-MSCs, with 5%–10% L-PIPL providing a bioactive environment that supports cytoskeletal organization and cartilage-associated matrix protein release.

**FIGURE 8:**
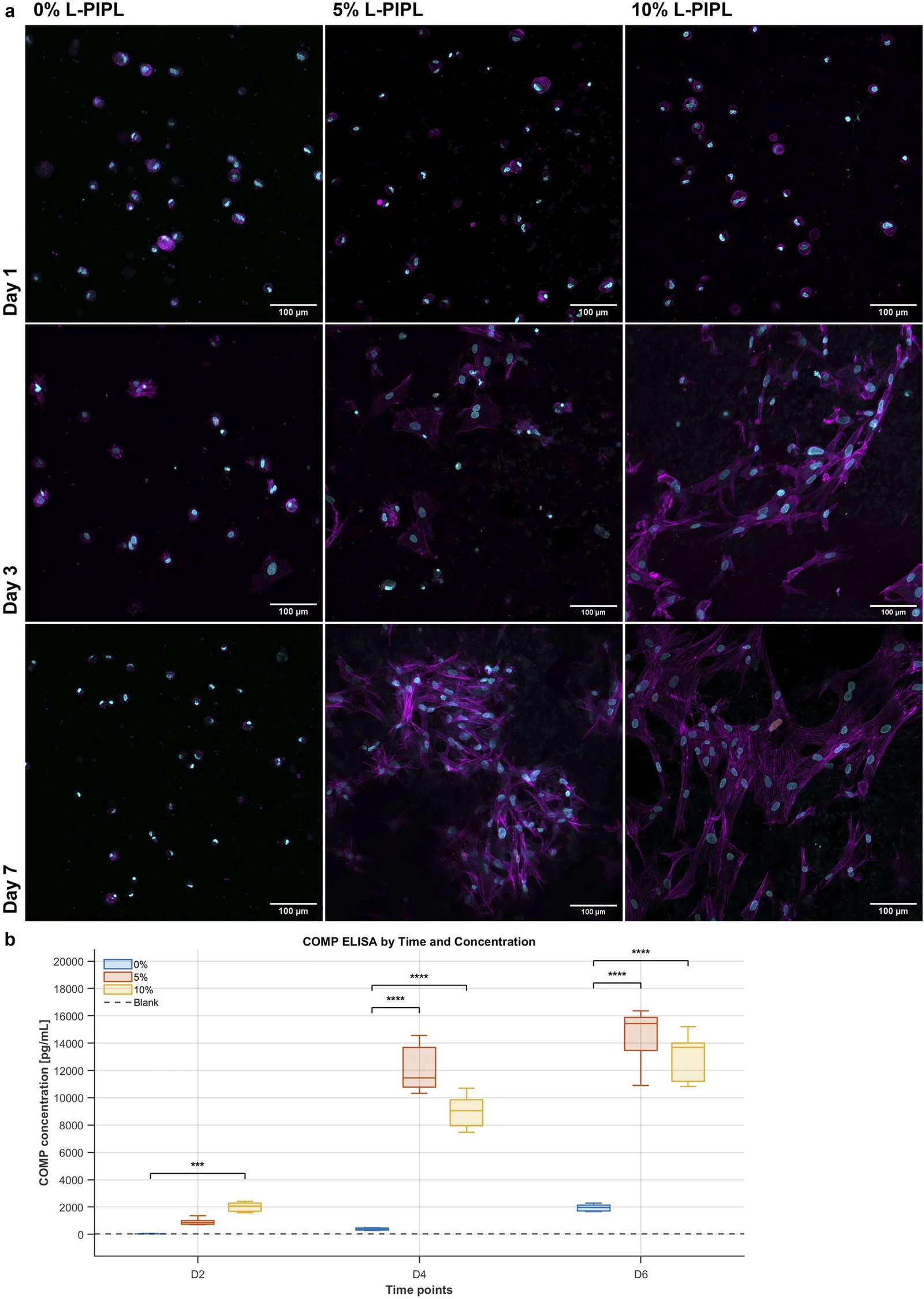
Early chondrogenic commitment of AD-MSCs encapsulated in L-PIPL-enriched hydrogels. **(a)** Representative immunofluorescence images of cells cultured in 0%, 5%, and 10% L-PIPL hydrogels at Days 1, 3, and 7. DNA is shown in cyan, F-actin in magenta, and SOX9 in red. Olympus FV1200 microscope; 20 × magnification; scale bar = 100 µm. **(b)** COMP concentration in the conditioned medium quantified by ELISA at Days 2, 4, and 6. The dashed line indicates the blank medium baseline. COMP data are presented as mean ± SD ( n=3 independent hydrogel constructs per condition). Statistical significance was assessed by two-way ANOVA followed by Bonferroni-adjusted post-hoc comparisons. Asterisks indicate Bonferroni-adjusted comparisons with the 0% L-PIPL control at the corresponding time point: * p<0.05 , ** p<0.01 , *** p<0.001 , **** p<0.0001 .

## 4 Discussion

This study demonstrates that incorporating lyophilized, pathogen-inactivated platelet lysate (L-PIPL) into a GelMA-based cartilage hydrogel yields a cytocompatible and bioactive 3D construct while preserving material features relevant for localized delivery. Beyond the general concept of platelet-derived enrichment, the novel contribution of this work is the use of an off-the-shelf L-PIPL format produced under a standardized Blood Bank workflow, together with a reproducible strategy to integrate L-PIPL into a defined commercial hydrogel and map how loading affects construct properties. This addresses a key translational limitation of platelet-based approaches, including biological variability and workflow dependence, which can drive inconsistent bioactivity and clinical performance Henschler et al. (2019); Zamani et al. (2019). In line with translational priorities, manufacturing and quality-control criteria for human platelet lysate (PL) have been emphasized to improve inter-batch and inter-center comparability Delabie et al. (2025); De Korte et al. (2024), and lyophilization has been proposed to enhance storage stability Wendland et al. (2025).

Across the evaluated formulations, moderate L-PIPL enrichment provided the most balanced overall profile. The 5%–10% (w/v) formulations maintained gel-like viscoelastic behavior and construct integrity while supporting high cytocompatibility and sustained release of TGF- β 1. TGF- β 1 was selected because of its central relevance to chondrogenic signaling Patil et al. (2011); Du et al. (2023); however, PL contains multiple bioactive factors, including PDGF and EGF, which may exhibit distinct release kinetics Burnouf et al. (2010); Marraccini et al. (2025). In addition, PL has been reported to promote chondrogenic differentiation of MSCs even in the absence of exogenous TGF- β supplementation Hassan et al. (2018), and mechanistic work has linked PL-induced chondrogenesis to regulation of the H19/miR-29b-3p/SOX9 axis Cao and Dai (2020). The qualitative SOX9-positive signal observed in the present study therefore supports the interpretation that L-PIPL enrichment provides platelet-derived cues associated with early chondrogenic commitment.

The biological responses observed here are consistent with previous cartilage tissue engineering studies using PL-based biomaterials, although the formulations and experimental settings differ considerably between studies. Teixeira et al. reported that PL incorporated into a dextran-based hydrogel promoted cell recruitment and chondrogenic responses in a cell-free cartilage-repair approach Teixeira et al. (2012), whereas Moroz et al. showed that platelet-lysate-based 3D scaffolds supported MSC chondrogenesis Moroz et al. (2013). Similarly, Jooybar et al. demonstrated that PL incorporated into an injectable hyaluronic-acid hydrogel supported the viability and chondrogenic matrix deposition of encapsulated MSCs Jooybar et al. (2019). Consistent with these studies, the present findings indicate that PL-derived bioactivity can be retained following incorporation into a hydrogel matrix, while further showing that both the biological and material responses depend on the amount of L-PIPL incorporated.

Although both 5% and 10% L-PIPL increased cell spreading and metabolic activity compared with the 0% control, the 5% formulation showed the most favorable early chondrogenic profile, as indicated by SOX9 immunofluorescence together with the highest COMP release at later time points. SOX9 is a nuclear transcription factor, and its immunofluorescence signal can be difficult to resolve clearly in 3D hydrogel constructs because it overlaps with the nuclear compartment; therefore, it should be interpreted as a qualitative indicator rather than a standalone quantitative endpoint. COMP is a cartilage-associated extracellular matrix protein involved in matrix organization and has been used as a secreted matrix-associated marker to quantify chondrogenic responses in MSC- and chondrocyte-based culture systems Uzieliene et al. (2023a); Uzieliene et al. (2023b). Its increased release supports an early cartilage-associated response rather than only increased general cell activity. In contrast, the 10% L-PIPL formulation induced stronger metabolic changes, including higher lactate accumulation and glucose consumption, but this was not matched by a proportional increase in COMP release or SOX9 expression. This suggests that higher L-PIPL concentrations may enhance cellular metabolic activity without necessarily improving lineage-specific chondrogenic commitment. Such uncoupling between proliferative/metabolic stimulation and chondrogenic outcomes is consistent with reports showing that human PL can enhance MSC proliferation while not necessarily improving chondrogenic differentiation outcomes Gardner et al. (2023). Similarly, the timing and context of PL supplementation has been shown to critically influence chondrogenic redifferentiation and matrix production Rikkers et al. (2020). Together, these findings support 5% L-PIPL as the most favorable condition in this study for the measured early chondrogenic readouts, whereas higher loading may shift the response toward increased metabolic activity rather than a stronger cartilage-associated response.

From a material perspective, ATR-FTIR showed increased protein-associated signals following L-PIPL incorporation without major changes in the characteristic chemical signature of the base hydrogel. Rheological and SEM analyses demonstrated that L-PIPL loading influenced matrix organization and mechanical behavior in a concentration-dependent manner. This is consistent with previous PL-containing hydrogel systems, in which the surrounding biomaterial provides the primary structural framework while PL acts as a bioactive component that can also modify the physical properties of the composite Teixeira et al. (2012); Jooybar et al. (2019). This differs from PRP/fibrin-based gels, where activation of plasma fibrinogen and formation of a fibrin network can contribute directly to hydrogel formation Wu et al. (2022). In the present system, moderate L-PIPL enrichment promoted a more porous and interconnected architecture, while higher loading altered network stability and viscoelastic behavior.

At the highest loading tested during the cytocompatibility screening, 15% L-PIPL reduced viability in cell-laden constructs. This response may reflect a combination of altered hydrogel microstructure and concentration-dependent biological effects of platelet-derived components rather than a single mechanism. Together with the rheological and microstructural changes observed at higher L-PIPL loading, these findings indicate that 15% L-PIPL is less suitable for the cell-laden application investigated here. However, higher-loading formulations may remain relevant for acellular applications, where the primary objective would be localized delivery of platelet-derived factors rather than supporting encapsulated cell survival Zhang P. et al. (2024); Tang et al. (2021).

Overall, L-PIPL enrichment provided a concentration-dependent balance between bioactivity and hydrogel performance. The 5%–10% (w/v) formulations maintained construct integrity and cytocompatibility, with 5% L-PIPL showing the most favorable early chondrogenic readouts and 10% L-PIPL producing stronger metabolic stimulation. In contrast, 15% L-PIPL reduced network stability and cell viability. These findings identify moderate L-PIPL loading as the most suitable range for further development of cell-laden cartilage constructs and indicate that increasing bioactive loading does not necessarily improve cartilage-associated responses.

The present study has some limitations, including the short duration of the biological assessment, the limited range of chondrogenic readouts, and the use of static *in vitro* culture conditions. Future work should therefore include longer *in vitro* experiments and a broader panel of chondrogenic markers to determine whether the early SOX9 and COMP responses translate into sustained cartilage matrix deposition and improved mechanical properties. Dynamic culture in bioreactor systems could provide a more physiological environment and help clarify whether nutrient transport and mechanical stimulation further enhance the performance of the 5% L-PIPL formulation. Additional studies using OA chondrocytes or inflammatory OA-like culture conditions would also be valuable to assess the anti-inflammatory and disease-modifying potential of the L-PIPL hydrogel. Although the present study primarily investigated L-PIPL as a bioactive matrix for encapsulated cells, a cell-free formulation could also be investigated as a localized depot for platelet-derived factors acting on surrounding resident cells. Finally, *ex vivo* wet adhesion testing and *in vivo* studies are needed to confirm safety, integration, retention, and regenerative efficacy in clinically relevant cartilage defect models. Collectively, these findings position L-PIPL-enriched hydrogels as a translationally oriented platform for localized delivery of platelet-derived cues and cartilage tissue engineering, with cell-laden regenerative constructs representing the primary application investigated here and cell-free injectable depots providing a potential direction for future development.

## Data Availability

The raw data supporting the conclusions of this article will be made available by the authors, without undue reservation.
