# Supplementary material for: Lyophilized pathogen-inactivated platelet lysate enriched hydrogel for cartilage tissue engineering
Source: Front Bioeng Biotechnol. 2026 Oct 27;14:1986446. doi: 10.3389/fbioe.2026.1986446 (PMC13748221; doi:10.3389/fbioe.2026.1986446)
Supplement: Supplementary file 1 [file Supplementaryfile1.docx]

# Supplementary material

Fig. S1. Live/Dead of AD-MSCs 24h after extrusion, before starting chondrogenic differentiation.

Fig. S2. D0 IF results with separate SOX9 channel.

Fig. S3. D3 IF results with separate SOX9 channel.

Fig. S4. D7 IF results with separate SOX9 channel.

| Assay | Time effect | Concentration effect | Interaction effect |
| --- | --- | --- | --- |
| Calcein-positive area | F(1,52)=63.95; p=1.28782e-10 | F(3,52)=18.01; p=3.81383e-08 | F(3,52)=10.62; p=1.48755e-05 |
| Mortality rate | F(1,52)=182.77; p=1.18396e-18 | F(3,52)=162.67; p=2.08268e-26 | F(3,52)=85.63; p=4.06203e-20 |
| ELISA TGF-β1 | F(6,56)=87.95; p=1.12074e-26 | F(3,56)=339.89; p=6.80317e-36 | F(18,56)=8.43; p=3.14539e-10 |
| Glucose levels | F(2,18)=3.88; p=0.0397 | F(2,18)=56.11; p<0.0001 | F(4,18)=1.06; p=0.4047 |
| Lactate levels | F(2,18)=0.92; p=0.4167 | F(2,18)=75.44; p<0.0001 | F(4,18)=1.42; p=0.2663 |
| Calcium levels | F(2,18)=108.68; p=8.94792e-11 | F(2,18)=59.01; p=1.24535e-08 | F(4,18)=18.78; p=3.11348e-06 |
| BCA | F(2,72)=109.60; p=1.4239e-22 | F(2,72)=94.65; p=7.02941e-21 | F(4,72)=19.02; p=1.03861e-10 |
| ELISA COMP | F(2,72)=518.82; p=1.72701e-43 | F(2,72)=516.92; p=1.95411e-43 | F(4,72)=96.33; p=3.9046e-28 |

Table S1. Two-way ANOVA results for the analyses performed.
